# Direct ink writing of tantalum: tailorable hierarchical porous scaffold for osteogenesis

**DOI:** 10.1093/nsr/nwag293

**Published:** 2026-05-26

**Authors:** Guangbin Zhao, Bochen Li, Ruiyan Liu, Zhenhua Zhou, Yuxin Gong, Lin Gao, Yating Nie, Xu Chen, Yanlong Wu, Xiaoxi Shao, Yichao Gong, Bo Li, Jianru Xiao, Yaxiong Liu, Bingheng Lu

**Affiliations:** State Key Laboratory for Manufacturing System Engineering, School of Mechanical Engineering, Xi’an Jiaotong University, Xi’an 710054, China; State Key Laboratory for Manufacturing System Engineering, School of Mechanical Engineering, Xi’an Jiaotong University, Xi’an 710054, China; School of Materials Science and Engineering, Xi’an University of Technology, Xi’an 710048, China; State Key Laboratory of Oral & Maxillofacial Reconstruction and Regeneration, National Clinical Research Center for Oral Diseases and Shaanxi Clinical Research Center for Oral Diseases, Department of Oral and Maxillofacial Surgery, School of Stomatology, The Fourth Military Medical University, Xi’an 710032, China; Department of Orthopaedic Oncology, Changzheng Hospital, Naval Military Medical University, Shanghai 200003, China; State Key Laboratory for Manufacturing System Engineering, School of Mechanical Engineering, Xi’an Jiaotong University, Xi’an 710054, China; State Key Laboratory for Manufacturing System Engineering, School of Mechanical Engineering, Xi’an Jiaotong University, Xi’an 710054, China; State Key Laboratory of Oral & Maxillofacial Reconstruction and Regeneration, National Clinical Research Center for Oral Diseases and Shaanxi Clinical Research Center for Oral Diseases, Department of Oral and Maxillofacial Surgery, School of Stomatology, The Fourth Military Medical University, Xi’an 710032, China; School of Mechatronic Engineering and Automation, Foshan University, Foshan 528000, China; School of Mechatronic Engineering and Automation, Foshan University, Foshan 528000, China; State Key Laboratory of Oral & Maxillofacial Reconstruction and Regeneration, National Clinical Research Center for Oral Diseases and Shaanxi Clinical Research Center for Oral Diseases, Department of Oral and Maxillofacial Surgery, School of Stomatology, The Fourth Military Medical University, Xi’an 710032, China; School of Materials Science and Engineering, Xi’an University of Technology, Xi’an 710048, China; State Key Laboratory for Manufacturing System Engineering, School of Mechanical Engineering, Xi’an Jiaotong University, Xi’an 710054, China; Department of Orthopaedic Oncology, Changzheng Hospital, Naval Military Medical University, Shanghai 200003, China; School of Mechatronic Engineering and Automation, Foshan University, Foshan 528000, China; State Key Laboratory for Manufacturing System Engineering, School of Mechanical Engineering, Xi’an Jiaotong University, Xi’an 710054, China

**Keywords:** direct ink writing, tantalum, bone implant, hierarchical porous metal, osteogenesis

## Abstract

To mitigate the risk of osseointegration failure in bone defect reconstruction, innovative fabrication strategies for developing implants with optimal biocompatibility and enhanced osteogenic capacity are crucial. Hierarchical porous structures formed by the synergistic combination of macropores (∼300–600 μm) and micropores (smaller than 20 μm) can better mimic the structural characteristics of native bone, thereby promoting osteogenesis. In this study, we developed direct ink writing (DIW) technology to fabricate a graded porous tantalum scaffold designed to promote osteogenesis. In the DIW printing process, a rheologically optimized ink and carefully calibrated printing parameters were utilized, enabling stable fabrication of patient-specific scaffold precursors with well-defined macropores. Following a controlled sintering process, the scaffolds exhibited macropores (∼400–500 μm in diameter) and interconnected micropores (∼0.5–23 μm in diameter), allowing tailoring of the mechanical properties and porosity to closely approximate those of native human bone. The well-controlled hierarchical porous structure exhibited excellent biocompatibility and significantly increased osteoinductive performance both *in vitro* and *in vivo*, accelerating new bone formation. We validated the reliable customization capabilities of this DIW-based tantalum scaffold printing method. The controllable porous structure further enhances the osseointegration potential of the implant, opening promising new avenues for the development of personalized bone implants.

## Teaser

The DIW-based approach enabled the printing of personalized tantalum scaffolds with hierarchical porosity, which significantly promoted new bone formation and osseointegration.

## INTRODUCTION

Bone defects caused by trauma, infection, bone tumours, and osteoporosis compromise the integrity of the skeletal system [1,2]. Autologous bone grafting for treatment of bone defects is often limited by donor shortages, secondary trauma, and infection risks. Customized porous scaffold implants, which are not subject to donor limitations and possess excellent load-bearing properties, hold promise as alternatives to autologous bone grafts [1,3]. An ideal implant necessitates materials that exhibit exceptional biocompatibility and osteogenic properties [4] coupled with a structure that replicates the hierarchical porous architecture of native bone [5,6]. Although titanium alloys are currently the most widely used materials for implants, their relatively insufficient osseointegration ability and risk of toxic metal ion (such as Al, V, and other toxic ions) release still limit optimization of clinical outcomes [6–9]. Recent advancements have demonstrated that porous tantalum, owing to its superior mechanical properties, corrosion resistance, biocompatibility, and inherent antimicrobial activity, is a promising material for bone implants [9–11].

Additive manufacturing (AM), in which a layer-by-layer fabrication strategy is employed, offers a promising solution for customized manufacturing of porous metal scaffolds [12–14]. Powder bed fusion (PBF) techniques, including selective laser melting (SLM) and electron beam melting (EBM), have emerged as mainstream processes in metal AM [15,16]. PBF technology offers considerable flexibility in designing and fabricating scaffolds with diverse structures and mechanical properties [6,17]. Studies have shown that hierarchical porosity can be achieved in PBF-fabricated scaffolds by adjusting energy input or scanning parameters, such as hatch spacing [18]. In practical applications, the use of hierarchical porous structures has not been widely explored due to concerns regarding mechanical stability [6,19,20].

Direct ink writing (DIW), through its unique room-temperature extrusion deposition mechanism [21], combined with controlled sintering processes, introduces open and interconnected microporous structures into scaffolds [22]. Owing to its excellent material adaptability, DIW has been widely applied in the fabrication of porous implants based on various metals and composite systems, including titanium [23], Ti-6Al-4V [24], high-entropy alloys [22], and Ti-TCP [25]. The printed macroporous structure allows adjustment of the mechanical properties over a broad range and meets personalized shape requirements for implants. Moreover, the scaffolds produced by DIW and subsequent sintering typically exhibit abundant microporosity while maintaining mechanical properties comparable to those of native bone and favourable biocompatibility [6]. The hierarchical porosity formed by the interaction of macropores and micropores in metal scaffolds better mimics the complex hierarchical structure of native bone tissue [5,6,26,27], not only matching the elastic modulus of the host bone to avoid stress shielding effects [2,22,28,29] but also creating three-dimensional channels that facilitate nutrient transfer and cell migration, significantly accelerating bone tissue ingrowth [5,22]. Nevertheless, to our knowledge, no studies have reported the fabrication of hierarchical porous tantalum scaffolds via DIW, and the potential of controllable sintering for achieving precise hierarchical pore regulation while synergistically optimizing mechanical and biological performance remains to be systematically explored.

**Figure 1.**
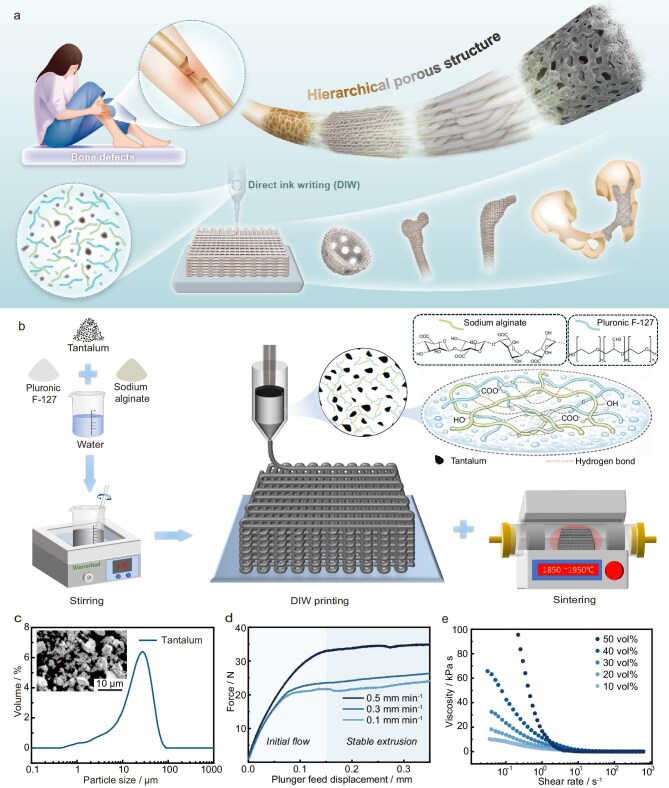
Hierarchical porous tantalum scaffolds fabricated by DIW printing. (a) Schematic illustration of porous tantalum scaffolds for osteogenesis. (b) Schematic of the porous tantalum scaffold fabrication process. The upper right corner shows a molecular-level schematic. (c) Particle size distribution of the tantalum powder (the figure on the upper left shows a scanning electron microscopy (SEM) image of the tantalum powder). (d) Force–displacement curves of the pre-printing ink subjected to extrusion processes at different plunger feed speeds. (e) Viscosity of inks with different solid loadings.

In this study, we utilized irregular tantalum powders to fabricate green scaffolds with high shape fidelity using DIW technology. Through a controlled sintering process, we achieved AM of hierarchical porous tantalum scaffolds with mechanical properties matching those of human bone and excellent biocompatibility. Specifically, a DIW ink formulation using Pluronic F-127 and sodium alginate as binders was developed (Fig. 1a), which exhibited good viscoelasticity and a high metal tantalum solid loading (40 vol%). This formulation enabled the printed green scaffolds to maintain their structural integrity and exhibit minimal shrinkage during sintering (<9%). Additionally, by systematically optimizing the DIW printing parameters (deposition height of 0.6 mm, plunger feed speed of 0.3 mm min^−1^, and nozzle moving speed of 20 mm s^−1^) and sintering conditions (temperature: 1850°C), the resulting hierarchical porous scaffolds exhibited mechanical properties comparable to those of cancellous bone (compressive strength: 90.46 ± 7.72 MPa; elastic modulus: 0.9 ± 0.31 GPa) while maintaining excellent biocompatibility and osteogenic potential. The macroporous architecture of these scaffolds provides ample space for bone ingrowth and vascularization [30], while the microporous structure further increases the specific surface area, creating an ideal microenvironment for bone cell migration, proliferation, and adhesion [31]. Its three-dimensionally interconnected and highly open pore system not only significantly increases the specific surface area of the scaffold but also effectively facilitates vascularization and new tissue ingrowth. Given the broad application prospects of such multiscale porous structures in fields requiring material flow or surface exchange, this study also provides a robust manufacturing approach for refractory metals such as tantalum in applications including catalysis [32,33], filtration [34,35], electrode materials [36,37], and heat exchangers [38,39].

## RESULTS AND DISCUSSION

### Optimizing ink rheology for enhanced printability

The DIW printing and sintering process for the porous tantalum scaffolds is shown in Fig. 1b. The printing ink was formulated by blending tantalum powder, Pluronic F-127 solution, and sodium alginate. The tantalum powder utilized in this study has an irregular morphology and a broad particle size distribution (Fig. 1c). This characteristic renders this approach more economically advantageous and easier to popularize than PBF techniques [40], which typically rely on spherical metal powders [41,42]. However, in the pre-printing ink formulation, the irregular powder cannot achieve the same uniform packing as spherical powders can, and the interparticle interactions are stronger [40,42]. Consequently, the ink should simultaneously maintain uniform metal particle dispersion and adequate flowability during printing. Pluronic F-127, a triblock copolymer composed of polyethylene oxide–polypropylene oxide–polyethylene oxide (PEO–PPO–PEO), exhibits liquid/gel transition behaviour near room temperature: its liquid state at lower temperatures enables homogeneous metal powder dispersion, while the gel state offers better rheological properties for DIW printing [43].

However, the high density of tantalum (16.69 g cm^−3^) increases the likelihood of sedimentation in the ink, thereby imposing more stringent stability requirements. Therefore, a thickener is necessary to improve the printability of the ink. Here, sodium alginate served as a thickener [44], with its carboxyl and hydroxyl groups interacting with the hydroxyl groups of Pluronic F-127 through hydrogen bonding (Fig. 1b). This interaction provides sufficient dynamic yield stress, enabling the ink to both counteract gravitational forces (weight of the whole printed structure), ensuring shape retention after printing, and support the following layers [44–46].

The printability of an ink is determined by its rheological properties; see Fig. 1d for the extrusion force of the ink at different plunger feed speeds. As the ink extrusion process progresses, the extrusion pressure gradually increases, resulting in the formation of two distinct phases: the initial flow phase and the stable extrusion phase. During the initial stage of extrusion, the extrusion pressure rapidly increases. At plunger feed speeds of 0.5, 0.3, and 0.1 mm min^−1^, the ink reaches a stable state after the feed distance reaches 0.15, 0.12, and 0.1 mm, respectively, which correspond to stabilization after extrusion for 18, 24, and 60 s. When the shear stress exceeds the yield stress of the material, the pre-printing ink begins to flow and quickly reaches a steady state, at which point the pressure curve stabilizes. This phenomenon can be attributed to the rheological properties of the pre-printing ink: Initially, the ink exhibits viscoelastic behaviour under compression, but once the pressure exceeds the critical stress, it yields and flows, resulting in viscoplastic behaviour [46].

As shown in Fig. 1e, all pre-printing materials with various solid loadings exhibit shear thinning effects. When the shear rate increases from 10^−1^ to 10^2^ s^−1^, the apparent viscosity decreases by several orders of magnitude. This rheological behaviour imparts excellent printability to the ink: when shear stress is applied, the viscosity of the material decreases, enabling it to flow under compression and yield. Conversely, the shear thinning is reversible: when the pressure is released, the viscosity rapidly increases, allowing the printed structure to maintain its shape [44]. Additionally, the solid loading of the ink also affects its rheological behaviour: an excessively high solid loading (for example, 50 vol%) leads to a high viscosity, and the ink may clog the nozzle, whereas an excessively low solid loading (<30 vol%) results in insufficient viscosity, making it difficult to maintain the structure [46]. Therefore, in this study, an ink with 40 vol% solid loading was selected for printing the porous tantalum scaffolds.

### DIW printing of porous tantalum green scaffolds

**Figure 2.**
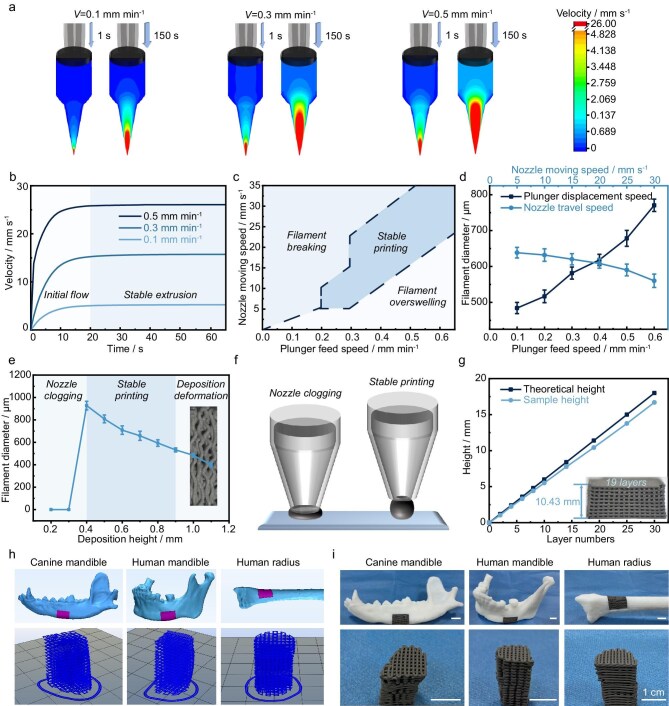
DIW with optimized process parameters facilitates the fabrication of customized porous tantalum green scaffolds. (a) Velocity distribution of the ink at different plunger feed speeds (0.1, 0.3, and 0.5 mm min^−1^), with the left side of each set of plunger feed speeds in the initial flow phase (loaded for 1 s) and the right side in the steady flow phase (loaded for 150 s) by CFD simulation. (b) Trend of the ink flow velocity at the centre of the nozzle outlet for different plunger feed speeds by CFD simulation. (c) Schematic of the printable area. (d) Different nozzle moving speeds and plunger feed speeds vs. filament diameter. (The nozzle speed was fixed at 20 mm s^−1^ when varying the plunger feed rate, and the plunger feed rate was fixed at 0.3 mm min^−1^ when varying the nozzle speed). (e) Deposition height vs. filament diameter. (f) Schematic of the effects of different deposition heights on the shape of the extruded ink. (g) Shape retention of the printed structure as a function of layer number (the figure on the lower right shows the scaffold with 19 printed layers). (h) 3D model design and (i) personalized porous tantalum scaffolds for canine mandibles, human mandibles, and human radii.

The fabrication of personalized green bodies via DIW is a fundamental prerequisite for manufacturing hierarchical porous tantalum scaffolds. The excellent ability to print the designed 3D patterns is influenced not only by the rheological properties of the ink but also by several factors, including the plunger feed speed, nozzle moving speed, and deposition height [47]. The computational fluid dynamics (CFD) simulation results demonstrate the ink flow velocity at the nozzle and provide theoretical guidance for process parameter optimization. The velocity distribution of the ink at different plunger feed speeds is shown in Fig. 2a; at any cross-section, the flow velocity at the centre is higher than that at the periphery, and the velocity at the centre progressively increases as the horizontal cross-sectional area decreases. The variation in the velocity at the centre point of the nozzle outlet under different plunger feed speeds is shown in Fig. 2b, revealing two distinct phases, namely, the initial flow phase and the stable extrusion phase, which are consistent with the experimental extrusion results (Fig. 1d). The CFD simulation results show that for feed rates of 0.5, 0.3, and 0.1 mm min^−1^, the ink reaches a steady state within 20 s in all the cases. While the simulation results for feed rates of 0.5 and 0.3 mm min^−1^ are in good agreement with the experimental data (18 and 24 s, respectively), the actual stabilization time for the 0.1 mm min^−1^ case (60 s) significantly deviates from the predicted value. This discrepancy may be attributed to liquid phase migration phenomena that occur at lower feed rates, under which the excessive outflow of the liquid phase during the initial extrusion stage leads to increases in the solid loading and viscosity of the subsequently extruded ink, thereby extending the time required to reach a stable extrusion state. This increase in ink viscosity due to liquid phase loss is similar to the difficulty in extruding ink from the nozzle in DIW printing with lignin, which has poor water retention [47]. Therefore, the plunger feed speed should be >0.1 mm min^−1^ to prevent the increase in the ink viscosity from affecting process control.

Stable and natural filament extrusion is important for DIW printing of porous tantalum green scaffolds; when the ink extrusion velocity matches the nozzle moving speed, the filament is deposited in a relatively natural manner [47]. The CFD simulations provide a theoretical reference for matching the printing process parameters. When the plunger feed speed is within the range of 0.3–0.5 mm min^−1^, the nozzle moving speed can be set in the range of 10–30 mm s^−1^ to ensure smooth filament deposition. The plunger feed speed and nozzle moving speed are a pair of synergistic parameters. Within the printable region (central zone of Fig. 2c), selection of appropriate printing parameters (for example, plunger feed speed and nozzle moving speed) enables ideal filament deposition. When the plunger feed speed is excessively high (lower-right zone of Fig. 2c) while the other parameters remain constant, the overaccumulation of ink at the deposition site per unit time leads to an excessive filament diameter (Fig. 2d) or even structural deformation. Conversely, if the nozzle moving speed is too high (upper-left zone of Fig. 2c), insufficient ink deposition per unit time causes excessive stretching or even breakage of the material.

The experimental data (Fig. 2d) demonstrate that under fixed parameters, the filament diameter is positively correlated with the plunger feed speed: when the speed increases from 0.3 to 0.6 mm min^−1^, the diameter increases from 581.50 ± 16.82 μm to 770.40 ± 17.17 μm, significantly deviating from the designed dimension (600 μm). Similarly, increasing the nozzle moving speed reduces the deposition cross-section because of the stretching effect, resulting in a decreased filament diameter; when the speed increases from 10 to 20 mm s^−1^, the diameter decreases from 631.98 ± 17.4 μm to 608.54 ± 13.23 μm, with the latter being closer to the target value. Therefore, the optimized printing parameter combination was determined to be a plunger feed speed of 0.3 mm min^−1^ and a nozzle moving speed of 20 mm s^−1^. This configuration balances the ink extrusion volume and printing efficiency, ensuring stable deposition while avoiding potential liquid-phase migration issues associated with low feed speeds (<0.1 mm min^−1^).

The layer-by-layer DIW manufacturing process for fabricating porous tantalum green scaffolds requires precise control of the deposition height (that is, the distance between the nozzle and the substrate), which is crucial for ensuring the structural integrity and dimensional accuracy of the scaffold. If the layer thickness is too large, then the contact between layers is reduced, leading to uneven material deposition, which negatively affects the strength of the scaffold. In contrast, if the layer thickness is too small, then the material will be excessively extruded, leading to deformation and affecting the pore size in the build direction. Therefore, the printing layer thickness was optimized. As shown in Fig. 2e, when the deposition height exceeds 0.9 mm, the extruded filament cannot immediately settle on the build platform, causing delay and distortion at the corners, and the material is stretched, leading to a reduction in the filament diameter (as shown in the right image of Fig. 2e). When the deposition height is <0.4 mm, the ink has a large contact area with the nozzle (as shown in the left image of Fig. 2f), leading to adhesion between the ink and the nozzle. The newly extruded material gradually accumulates at the nozzle, causing blockage and preventing proper material extrusion. When the deposition height is in the range of 0.4–0.9 mm, although the ink printing is stable, significant deformation of the filaments occurs if the deposition height deviates too much from the nozzle diameter (600 μm). Only when the deposition height is close to the nozzle diameter does the material avoid excessive compression or stretching (as shown in the right image of Fig. 2f), resulting in the best deposition result. Therefore, the final printing parameters determined through single-layer experiments were a deposition height of 0.6 mm, a plunger feed speed of 0.3 mm min^−1^, and a nozzle moving speed of 20 mm s^−1^.

We systematically evaluated the shape retention capability of 3D-printed porous tantalum green scaffolds, with a particular emphasis on the build direction in which filament collapse is most likely to occur. After drying, the porous tantalum green scaffolds maintain excellent structural integrity with only minimal deformation (Fig. 2g). For this printing experiment, the single-layer filament thickness was set at 0.6 mm. As shown in Fig. 2g, the printed green scaffolds are close to their theoretical heights: at 2, 6, 10, and 19 layers, the measured thicknesses are 1.04, 3.32, 5.51, and 10.43 mm, respectively. Notably, the sample height demonstrates an approximately linear correlation with the number of printed layers, and the ratio of the actual height to the theoretical height remains nearly constant (∼90.7%). These findings indicate that the porous tantalum green scaffolds exhibit outstanding structural stability during both the printing and post-printing stages, effectively preventing collapse. Through optimized structural design, we achieved high-fidelity printing of porous tantalum green scaffolds with precisely controlled thicknesses.

The optimized printing stability combined with the exceptional shape retention characteristics of the green scaffolds enables precise manufacturing of personalized porous tantalum scaffolds. We designed (Fig. 2h) and fabricated (Fig. 2i) customized porous tantalum green scaffolds for bone defect models (canine mandible, human mandible, and human radius). As shown in Fig. 2i, the fabricated personalized green scaffolds exhibit geometric morphology with high congruence with the host bone tissue, further confirming that the DIW fabrication process adopted in this study fulfils the requirements for morphological customization of porous tantalum implants. Moreover, the superior process controllability of this technique provides substantial design flexibility for scaffold architectures, enabling potential structural optimizations to balance biocompatibility and mechanical performance, thereby ensuring long-term functional and structural integrity in clinical applications [6].

Although the ink developed in this study exhibited favourable rheological properties, enabling reliable printability and structural stability in DIW, certain limitations remain when compared with the well-established SLM technique widely used for clinical fabrication of porous tantalum scaffolds [48]. The primary advantage of SLM is its high design flexibility, allowing the fabrication of complex geometries while maintaining excellent dimensional accuracy and resolution [49]. In contrast, DIW is constrained by the nozzle diameter, which imposes a lower limit on filament width, and the relatively lower resolution of DIW further limits its flexibility in structural design [21]. Moreover, as the number of printed layers increases, deviations between the actual and theoretical scaffold height in the *Z* direction tend to accumulate, thus affecting overall structural precision [22].

### Tailorable hierarchical porosity and mechanical behaviour

**Figure 3.**
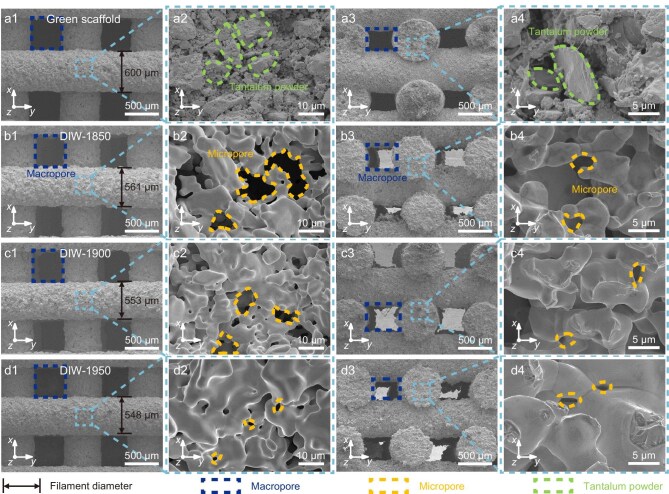
SEM images of the green scaffolds and sintered scaffolds. (a) Green scaffold and scaffolds sintered at (b) 1850°C, (c) 1900°C, and (d) 1950°C, showing their (1 and 2) surface and (3 and 4) cross-sectional morphologies. The rectangular dashed lines in the macroscopic images of the scaffolds (1 and 3) indicate macropores; the irregular dashed lines in the microscopic morphology of the green scaffold (a2 and a4) indicate tantalum powder; and the irregular dashed lines in the microscopic morphology of the sintered scaffolds (b2, b4, c2, c4, d2, and d4) indicate micropores.

The morphologies of the porous tantalum green scaffolds in both horizontal (Fig. 3a1 and a2) and vertical (Fig. 3a3 and a4) cross-sections were observed. The structural integrity of the green scaffolds is maintained, with well-defined macroporous architectures and defect-free interlayer interfaces. The filament diameters remain consistent at 600 ± 15 μm, with a uniform morphology, showing no significant deformation. Crucially, the binder phase forms a homogeneous coating around the tantalum particles (Fig. 3a2 and a4), while the particles themselves are evenly distributed within the filament structure. This uniform microstructure ensures controlled binder removal during debinding, facilitating the formation of uniformly distributed, interconnected micropores during subsequent sintering.

The macroscopic morphology and vertical surface topography of the scaffolds sintered at various temperatures are shown in Fig. 3b–d. Following sintering, the filament diameter is ∼554 μm, and a uniform and intact cylindrical geometry is maintained. Owing to their isotropic and homogeneous shrinkage characteristics, the scaffolds preserve their three-dimensional structural integrity without warping or cracking defects while macropores remain distinctly discernible (Fig. 3b1, c1, and d1). Microstructural analysis reveals that the tantalum particles achieve metallurgical bonding through well-developed sintering necks. In contrast to dense metallic scaffolds fabricated via PBF [14], the surfaces of the present tantalum scaffolds (Fig. 3b2, c2, and d2) exhibit neither unmolten particles nor impurity phases; instead, uniformly distributed microporous structures are formed. Similarly, interconnected micropores are observed within the scaffold cross-sections (Fig. 3b4, c4, and d4). These micropores likely originate from volatilization of organic components during pyrolysis, coupled with incomplete void filling by the liquid phase during metallic sintering. This mechanism facilitates the formation of three-dimensionally interconnected open pores, thereby effectively increasing the specific surface area of the scaffold. Notably, as the sintering temperature increases, the size of the micropores significantly decreases (Supplementary Material 1). At a sintering temperature of 1850°C, the micropores are relatively large, with diameters ranging from 1 to 23 μm (D90 = 20.4; D90 refers to the pore diameter below which 90% of the measured pores are smaller). As the temperature increases to 1900°C, the pore size range decreases to 0.75–12.7 μm (D90 = 11.05). Furthermore, at a higher sintering temperature of 1950°C, the pore size range narrows even further to 0.5–7.2 μm (D90 = 5.55). These findings indicate that the formation and distribution of micropores can be regulated through precise control of the sintering parameters. The hierarchical porous structure, engineered through a combination of macropores (∼400–500 μm in diameter) and sintering micropores (∼0.5–23 μm in diameter), yields a substantial surface area conducive to bone growth. This hierarchical porosity (involving pore size and structural variations) facilitates mechanical interlocking between bone and the implant, which is a mechanism widely recognized for enhancing implant fixation [26]. Furthermore, the highly interconnected porous network provides optimal pathways for the transport of nutrients and fluids, thereby fostering bone ingrowth and promoting long-term mechanical stability [5,6].

**Figure 4.**
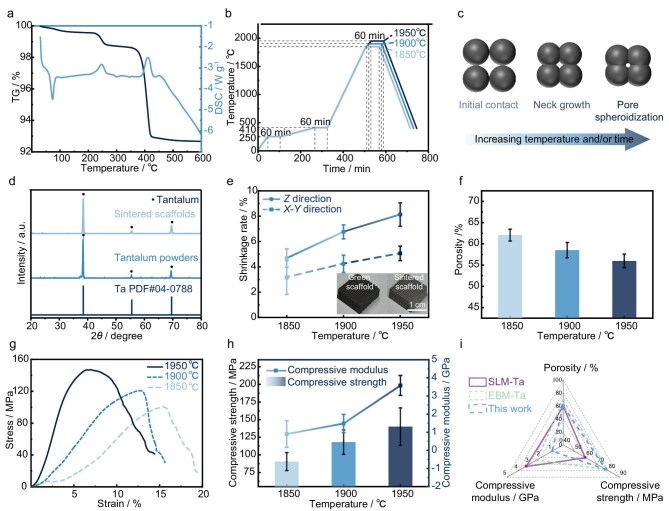
Sintering process and mechanical properties of the porous tantalum scaffolds. (a) Differential scanning calorimetry (DSC)-thermogravimetric (TG) curves of the green scaffolds. (b) Sintering schedules investigated, with holding at 1850°C, 1900°C, or 1950°C for 1 h. (c) Schematic of metal powder sintering. (d) XRD results of the raw tantalum powder and sintered scaffolds. (e) Shrinkage rate, (f) open porosity, (g) stress–strain curve, (h) compressive strength and modulus of the porous tantalum scaffolds sintered under different temperatures. (i) Radar plot benchmarking the key performance parameters (porosity, compressive strength and modulus) of the porous tantalum scaffolds fabricated in this work and other metal AM technologies (SLM-Ta and EBM-Ta represent porous tantalum scaffolds prepared by SLM and EBM technologies, respectively) [50].

To investigate the changes in composition of the scaffolds during sintering, thermogravimetric analysis was performed on the green scaffolds at a heating rate of 1°C min^−1^, and the resulting curve is shown in Fig. 4a. The initial weight loss (60–140°C) corresponds to the evaporation of residual free water and bound water within the sodium alginate matrix. Organic decomposition occurs in two distinct stages: the first between 230 and 270°C (peak at 250°C) and the second between 380 and 430°C (peak at 410°C). Notably, pyrolysis of organics typically generates substantial amounts of gaseous byproducts, which may induce crack formation or structural damage because of rapid gas release [50,51]. To mitigate this, in the debinding process, lower heating rates and extended dwell times were employed during pyrolysis (Fig. 4b), effectively moderating the gas evolution rates. Following complete decomposition and controlled gas release, the interconnected channels formed within the structure establish a prerequisite for uniform micropore formation [52]. Post-debinding (above 410°C), the sintering stage commences. As illustrated in Fig. 4c, metallic sintering progresses through three characteristic phases: initial contact, neck growth, and pore spheroidization [53]. In this study, the sintering temperature served as the primary control parameter (Fig. 4b) to regulate the shrinkage rates, porosity characteristics, and mechanical properties. Upon completion of sintering, the tantalum scaffolds underwent furnace cooling to minimize residual thermal stresses. The X-ray diffraction (XRD) patterns of the raw powders and sintered scaffolds are shown in Fig. 4d, and all the patterns exhibit characteristic peaks corresponding to pure tantalum (PDF No. 04-0788). These results indicate that the crystalline phase of the sintered scaffolds is the same as that of the raw tantalum powder (Fig. 1c), and there are no other noticeable impurities in the sintered scaffolds. Compared with PBF technology involving rapid metal powder melting and cooling, the combination of DIW with controlled and relatively moderate sintering processes eliminates unmelted powders or impurities on scaffold surfaces that could compromise the mechanical performance [20,54].

The shrinkage of the scaffolds in the building and horizontal directions after sintering at different temperatures is shown in Fig. 4e. Owing to the influence of gravity, the shrinkage in the building direction is slightly greater than that in the horizontal direction after sintering. As the sintering temperature increases, the shrinkage rate in both directions gradually increases, which may indicate that a higher sintering temperature leads to increased densification of the scaffold. Compared with the porosity (∼40%) of hierarchical porous scaffolds made from green scaffolds containing metal oxides by reduction sintering [51], the shrinkage rate of the hierarchical porous tantalum scaffolds produced in this study (<10%) is significantly lower. The lower shrinkage rate allows a more rational design of the structure and sintering process and reduces stresses during sintering, preventing potential warping and cracking [55], thereby better meeting the requirements of personalized bone implant manufacturing. Additionally, increasing the sintering temperature progressively reduces the scaffold porosity (Fig. 4f). The porosity of the hierarchical porous tantalum scaffolds sintered at 1850°C is 62.07 ± 1.39%, which decreases to 55.98 ± 1.61% when the sintering temperature is increased to 1950°C. These results further demonstrate that higher sintering temperatures enhance scaffold densification while enabling effective control over micropores through temperature regulation.

Evidence indicates that maintaining a modulus comparable to that of natural bone tissue is crucial for ensuring effective load distribution and long-term osseointegration in load-bearing applications [6,28,29]. However, the modulus of bone (cancellous bone: 0.04–3.3 GPa) significantly varies depending on age, anatomical site, and bone quality [56,57]. Therefore, the mechanical properties of bone implants must be balanced according to practical requirements. The stress–strain curves of hierarchical porous tantalum scaffolds sintered at different temperatures are shown in Fig. 4g. The results demonstrate that scaffolds sintered at higher temperatures exhibit lower failure strain but higher compressive strength, indicating an increase in the compressive modulus. When the sintering temperature is increased from 1850 to 1950°C, the compressive strength of the scaffolds increases from 90.46 ± 7.72 to 140.07 ± 9.6 MPa, and the compressive modulus increases from 0.9 ± 0.31 to 3.58 ± 0.49 GPa (Fig. 4h). The compressive strength and elastic modulus are negatively correlated with increases in porosity, consistent with the trend predicted by the Gibson–Ashby model for stress variation with porosity [58].

This controllable sintering process allows the mechanical properties of hierarchical porous tantalum scaffolds to be adjusted within a specific range, enabling precise matching with the mechanical requirements for different bone implants. At similar porosity levels (∼60%), the hierarchical porous tantalum scaffolds fabricated in this study exhibit compressive strengths comparable to those of scaffolds produced via EBM technology, while their compressive moduli are closer to that of natural bone than to those of scaffolds fabricated by EBM and SLM (Fig. 4i) [48]. This better match with the stiffness of bone tissue can effectively reduce bone loss [59]. More importantly, the reduced stiffness enhances load-sharing with surrounding bone, thereby decreasing the risks of stress shielding and implant loosening. Furthermore, the hierarchical porous design improves the stress distribution between the implant and peri-implant bone, which further enhances the long-term stability while reducing the risk of implant loosening [6].

### Cytocompatibility and osteogenic assessment

**Figure 5.**
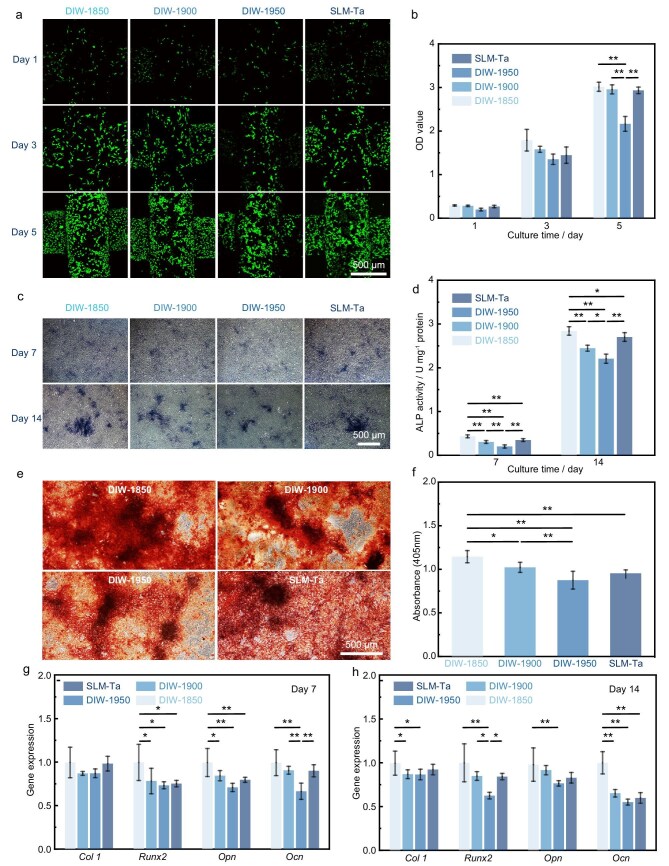
*In vitro* osteogenic performance of porous tantalum scaffolds. (a) Live/dead staining images of BMSCs cultured on scaffolds on days 1, 3, and 5. (b) Quantitative analysis of cell proliferation via a CCK-8 assay (OD values) on days 1, 3, and 5. (c) ALP staining images on days 7 and 14. (d) Quantification of ALP activity. (e) Alizarin red S staining for mineralized matrix on day 21. (f) Quantitative analysis of alizarin red S staining on day 21. (g) Expression levels of osteogenesis-related genes (collagen type I *(Col 1)*, Runt-related transcription factor 2 *(Runx2)*, osteopontin *(Opn)*, and osteocalcin *(Ocn)*) on day 7. (h) Gene expression analysis of *Col 1, Runx2, Opn*, and *Ocn* on day 14. The data are presented as the mean ± standard deviation (*P < 0.05; **P < 0.01).

To validate the cytocompatibility and *in vitro* osteogenic performance of the scaffolds, we investigated the effects of scaffolds sintered at different temperatures on the adhesion, proliferation, and mineralization behaviour of bone marrow mesenchymal stem cells (BMSCs). The effects of scaffolds sintered at different temperatures on BMSC adhesion and proliferation are shown in Fig. 5a and b. The live/dead staining results (Fig. 5a) reveal progressive increases in the viable cell density on all the scaffolds over extended culture periods. Scaffolds sintered at 1850°C consistently maintain elevated live cell densities at days 1, 3, and 5. Correspondingly, the CCK-8 assay data (Fig. 5b) demonstrate concordant trends, with the optical density (OD) values increasing in a time-dependent manner. Compared with the comparative groups, the DIW-1850 group showed higher OD values than the other groups, with significant differences observed at day 5 (*P* < 0.05). These findings reveal favourable cytocompatibility across all four scaffold formulations in terms of supporting BMSC adhesion and proliferation, with the 1850°C-sintered scaffolds demonstrating superior enhanced adhesion and proliferation capacity throughout the observation window. The SLM-Ta scaffold developed molten tracks on its surface during sintering, resulting in a relatively smooth surface lacking micropores. This configuration yields a lower specific surface area than that of scaffolds with a hierarchical porosity.

Consequently, this scaffold is less effective at promoting BMSC adhesion than DIW-1850, and it does not exhibit a significantly inferior adhesion-promoting capacity compared with the DIW-1900 and DIW-1950 scaffolds. This is attributable to the excessively small micropores of the DIW-1900 and DIW-1950 scaffolds that easily induce early pore occlusion after cell seeding, which offsets the theoretical advantage of their hierarchical porous structure and results in a cell adhesion level comparable to that of the SLM-Ta scaffold [63,64]. In contrast, the larger, moderate micropore size of the DIW-1850 scaffold avoids premature pore blockage, facilitating more efficient nutrient exchange with increasing pore size, and its hierarchical porous structure synergistically provides abundant adhesion sites, thus demonstrating a superior ability to promote BMSC adhesion.

The *in vitro* osteogenic differentiation of BMSCs on scaffolds sintered at different temperatures is shown in Fig. 5c–f. ALP staining (Fig. 5c) reveals positive staining across all four scaffold groups, with the ALP activity levels progressively increasing over time. Quantitative analysis of the alkaline phosphatase (ALP) activity (Fig. 5d) reveals significantly higher ALP activity levels in the DIW-1850 group than in the other groups on both days 7 and 14 (*P* < 0.05). Alizarin red S staining on day 21 (Fig. 5e) confirms mineralized nodule formation in all groups, with the DIW-1850 group exhibiting more numerous and larger calcified deposits. Quantitative analysis (Fig. 5f) demonstrates that DIW-1850 induces the greatest degree of mineral deposition (*P* < 0.05). Analysis of osteogenic marker genes (Fig. 5g–h) showed that the DIW-1850 group generally exhibited higher expression levels of *Col 1, Runx2, Opn*, and *Ocn* than the other groups, with significant differences observed for several markers on days 7 and 14 (*P* < 0.05). These findings demonstrate that the scaffolds sintered at 1850°C exhibit superior *in vitro* osteogenic performance.

The DIW-1850 scaffold, with its larger micropore dimensions (∼0.5–23 μm in diameter) and higher porosity (62.07 ± 1.39%), has a greater specific surface area, consequently offering more sites for cell adhesion and promoting pseudopod extension. This cell adhesion is functionally coupled to osteogenesis [31,62]. The proadhesive effect also promotes cell proliferation and greater cell–cell contact. This increased contact coordinates the formation of complex intercellular signalling networks on the DIW-1850 scaffold, augmenting both juxtacrine and paracrine communication [63]. These pathways activate local signalosomes, driving osteoblastic lineage commitment in BMSCs [64,65], as molecularly substantiated by the upregulated expression of osteogenic markers in this study. Through the secretion of the extracellular matrix by differentiated BMSCs and the provision of an increased surface area by the larger micropores and the greater porosity, the attachment of osteoinductive biomolecules and the precipitation of biological apatite are enhanced, accelerating *in vitro* mineralization [30,64]. The expanded micropore network and high porosity increase the permeability of the scaffold, facilitating the diffusion of oxygen, nutrients, and growth factors to cells within the scaffold [6,66,67] and thus promoting cell proliferation and growth *in vitro*. These findings demonstrate that the structure of the DIW-1850 scaffold creates a highly favourable microenvironment that promotes cellular processes essential for adhesion, proliferation, and mineralization.

### *In vivo* osteogenesis evaluation

**Figure 6.**
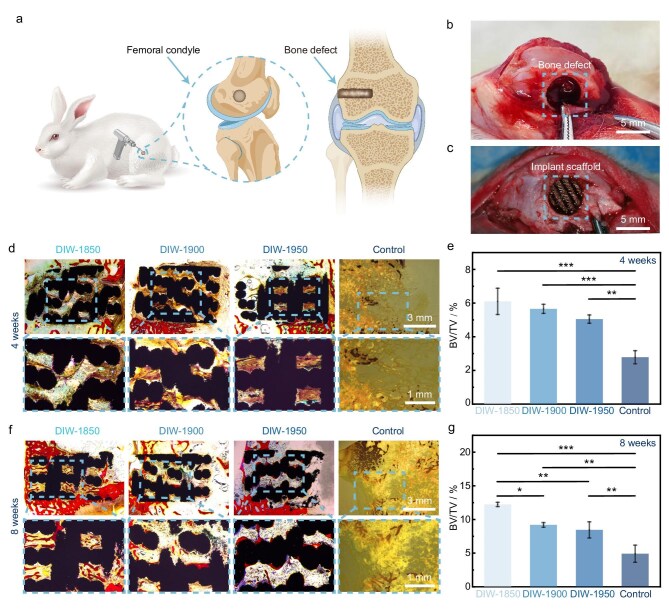
*In vivo* bone repair performance of porous tantalum scaffolds. (a) Defect site in a rabbit lateral femoral condyle. (b) Bone defect cavity. (c) Scaffold implantation into the defect site. (d and f) Van Gieson (VG) staining of femoral condyle defects at (d) 4 weeks and (f) 8 weeks post-implantation for the DIW-1850, DIW-1900, DIW-1950, and blank control groups. (e and g) Bone volume fractions (BV/TV) of newly formed bone at (e) 4 weeks and (g) 8 weeks for scaffolds fabricated at different sintering temperatures. The data are presented as the mean ± standard deviation (*P < 0.05; **P < 0.01; ***P < 0.001).

Following the creation of bone defect models in the lateral femoral condyle of rabbits, the scaffolds were implanted into the bone defects (Fig. 6a–c). The results of the *in vivo* experiments are shown in Fig. 6d and f. The results reveal no signs of inflammation in any animal, and the DIW-1850 group consistently exhibits the most extensive new bone formation at both time points: At week 4, bone tissue begins to form around the scaffold; by week 8, new bone has largely filled the pore regions, forming continuous bridging structures with indistinct scaffold–bone boundaries, indicating strong osseointegration and tissue maturation. In contrast, new bone in the DIW-1900 group is primarily localized to the scaffold periphery, with insufficient ingrowth in the central region. In the DIW-1950 group, bone formation is limited, and the pores are mostly occupied by soft tissue. The blank control group exhibits primarily fibrous tissue with poor bone repair, underscoring the critical role of the scaffold in osteogenesis.

To further evaluate bone regeneration, the bone volume to total volume fraction (BV/TV) was quantitatively analysed at weeks 4 and 8 (Fig. 6e and g). The results reveal that the BV/TV values of all the scaffold groups increase at 8 weeks compared with those at 4 weeks. Notably, the DIW-1850 group exhibits the greatest increase (ΔBV/TV = 6.14%), whereas the DIW-1900 and DIW-1950 groups show increases of 3.54% and 3.41%, respectively. In contrast, the blank group demonstrates only a 2.13% increase. These findings further confirm the sustained, stable, and significant osteogenic advantage of the DIW-1850 scaffold during the bone formation process.

The larger micropores and greater porosity of DIW-1850 scaffolds enhance cell attachment, migration, and proliferation, thus promoting early-stage osseointegration [30,68]. Micropore-induced capillary forces recruit biomolecules from physiological fluids at the defect site and distribute them throughout the scaffold interior [69,70]. This process facilitates biological apatite deposition on the scaffold surface, thus accelerating *in vivo* biomineralization [69]. Micropores serve as physical compartments for microscale bone growth [69,71], creating an interconnected space for bone tissue ingrowth. The expanded micropore network and high porosity facilitate capillary ingrowth [30,72], establishing advanced nutrient transport routes that accelerate the bone healing cascade.

## CONCLUSION

In summary, we have successfully fabricated tantalum scaffolds with a tailorable hierarchical porosity by DIW with precisely controlled sintering. The ink exhibits excellent rheological properties and demonstrates remarkable printability and shape fidelity. These characteristics facilitate stable fabrication of green bodies with well-defined macropores, fulfilling the morphological customization requirements essential for bone implants. In contrast to high-energy melting processes, controlled sintering enables the formation of both macropores (∼400–500 μm in diameter) and interconnected micropores (∼0.5–23 μm in diameter), a feature that is challenging to achieve via PBF, thereby establishing favourable conditions for long-term implant stability and osseointegration. Specifically, the porosity of the scaffold sintered at 1850°C is 62.07 ± 1.39%, and the compressive strength (90.46 ± 7.72 MPa) and elastic modulus (0.9 ± 0.31 GPa) are within the ranges reported for human cancellous bone. As evidenced by both *in vitro* and *in vivo* studies, the scaffold sintered at 1850°C exhibits excellent performance in terms of various critical parameters, including cell proliferation, differentiation, osteogenesis, and bone ingrowth. Sintering parameters significantly influence the microstructure and mechanical properties of tantalum scaffolds. While larger micropores and greater porosity facilitate nutrient transport, cell infiltration, and osteogenic differentiation, excessive porosity may compromise the mechanical strength of the scaffold. Controlled sintering provides a stable and reliable approach to balancing mechanical performance with biocompatibility.

Despite the slightly lower printing resolution and precision of DIW compared with SLM, the unique hierarchical porosity generated through DIW combined with controlled sintering substantially enhances the osteogenic performance of the implants, providing strong support for the broader application of porous tantalum scaffolds. Notably, our approach circumvents the necessity for costly spherical powders; the compatibility with irregular powders substantially reduces material expenses and improves clinical accessibility, further facilitating translational potential. Moreover, this method presents a promising, cost-effective, and adaptable printing technique for refractory metals with hierarchical porous structure, offering potential for structural and material optimization in applications requiring high material flow or efficient surface exchange, such as catalysis and filtration. Future research will focus on expanding the range of printable metals, enhancing printing precision, and optimizing processing parameters and structural designs for diverse applications.

## MATERIALS AND METHODS

### Ink preparation

Tantalum powder (99.9% purity; Qinghe Jiechuang Metal Products Co., Ltd., China) with an average particle size of 24.19 μm was used in this study. Sodium alginate powder (Shanghai Sangon Biotech Co., Ltd., China) and Pluronic F-127 (Shanghai Macklin Biochemical Technology Co., Ltd., China) were selected as binders.

Preparation of printing ink: specified amounts of sodium alginate (5.0 wt%) and Pluronic F-127 (20 wt%) were dissolved in deionized water and then stirred in an ice–water bath until a homogeneous paste was obtained. Tantalum powder was incrementally introduced into the mixed solution under sustained stirring until complete homogenization was achieved, thereby yielding inks with suitable rheological characteristics and mechanical stability. The rheological property testing of the ink and the CFD simulation method are provided in Supplementary Material 2.

### DIW printing and sintering

The ink was placed in a barrel for loading into a DIW printer (homemade, with a nozzle diameter of 600 μm). The motor-driven plunger pressurized and extruded the ink through the nozzle, enabling layer-by-layer deposition on a 3D platform to form the final model. During the fabrication of 3D porous scaffolds, the deposition height was found to critically influence the printing quality. Systematic optimization was performed by printing single-layer patterns, with the deposition height incrementally adjusted from 0.2 to 1.1 mm at 0.1-mm intervals to determine the optimal layer thickness. Following layer thickness determination, a parametric study was conducted using six plunger feed rates (0.1–0.6 mm min^−1^, 0.1 mm min^−1^ increments) and six nozzle moving speeds (5–30 mm s^−1^, 5 mm s^−1^ increments) in pairwise combinations for single-layer deposition. The printing quality was subsequently observed, and the filament diameter was measured using a Vernier calliper to select the appropriate range of process parameters and determine the optimal combination of plunger feed speed and nozzle movement speed.

3D porous scaffolds were printed with an overall size of 21.85 × 21.85 × 9 mm^3^, a filament diameter of 600 μm, a spacing of 600 μm and filaments of adjacent layers crossing at 90°. The printed porous tantalum green scaffolds were subsequently dried in a vacuum oven (DZF-6020; Shanghai Shanzhi Instrument Equipment Co., Ltd., China) at 50°C for 5 h.

After that, the dried porous tantalum green scaffolds were placed in a high-temperature sintering furnace (KCE-FP W 6 LA/BL, FCT System GmbH, Germany) for debinding and sintering in a vacuum environment (pressure below 8 × 10^−2^ Pa). In the debinding stage, the temperature was increased from room temperature to 250°C at 5°C min^−1^, held for 60 min, then increased to 410°C at 1°C min^−1^, and held for 60 min. Subsequently, the temperature in the furnace was increased to different sintering temperatures at 8°C min^−1^, held for 1 h, and finally cooled; these samples were labelled DIW-1850, DIW-1900, and DIW-1950, where DIW represents porous tantalum scaffolds prepared by DIW technology, and the number represents the sintering temperature. The characterization methods are described in Supplementary Material 3.

The cytocompatibility and osteogenic capacity of the scaffolds were evaluated through *in vivo* and *in vitro* biological experiments. The characterization methods of the scaffolds and the testing methods for osteogenic performance are provided in Supplementary Material 4.

### Statistical analysis

Data are expressed as the mean ± standard deviation and were analyzed using SPSS version 19.0. Comparisons among multiple independent groups were performed using one-way analysis of variance followed by Tukey’s post-hoc test. A value of *P* < 0.05 was considered statistically significant.

## Supplementary Material

nwag293_Supplemental_File
