# Supplementary material for: Direct ink writing of tantalum: tailorable hierarchical porous scaffold for osteogenesis
Source: Natl Sci Rev. 2026 May 26;13(13):nwag293. doi: 10.1093/nsr/nwag293 (PMC13348250; doi:10.1093/nsr/nwag293)
Supplement: nwag293_Supplemental_File [file nwag293_supplemental_file.pdf]

Supplementary information file

Direct ink writing of tantalum: tailorable hierarchical porous scaffold for osteogenesis

Guangbin Zhao[1,†], Bochen Li[1,2,†], Ruiyan Liu[3,†], Zhenhua Zhou[4], Yuxin Gong[1], Lin Gao[1], Yating Nie[3], Xu Chen[5], Yanlong Wu[5], Xiaoxi Shao[3,*], Yichao Gong[2,*], Bo Li[1,*], Jianru Xiao[4,*], Yaxiong Liu[5] and Bingheng Lu[1]

[1]State Key Laboratory for Manufacturing System Engineering, School of Mechanical Engineering, Xi'an Jiaotong University, Xi'an 710054, China;
[2]School of Materials Science and Engineering, Xi'an University of Technology, Xi'an 710048, China;
[3]State Key Laboratory of Military Stomatology and National Clinical Research Center for Oral Diseases and Shaanxi Clinical Research Center for Oral Diseases, Department of Oral and Maxillofacial Surgery, School of Stomatology, The Fourth Military Medical University, Xi'an 710032, China;
[4]Department of Orthopaedic Oncology, Changzheng Hospital, Naval Military Medical University, Shanghai 200003, China;
[5]School of Mechatronic Engineering and Automation, Foshan University, Foshan 528000, China.
[*]Corresponding authors. E-mails: sxxi77@163.com; gongyichao@xaut.edu.cn; liboxjtu@xjtu.edu.cn; jianruxiao83@163.com
[†]Equally contributed to this work.

Contents

Supplementary Material 1: Pore size distributions of porous tantalum scaffolds..........2
Supplementary Material 2: Computational fluid dynamics (CFD) simulation..............3
Supplementary Material 3: Characterization 4
Supplementary Material 4: Cytocompatibility and osteogenic assessment...................5
REFERENCES 8

Supplementary Material 1: Pore size distributions of porous tantalum scaffolds

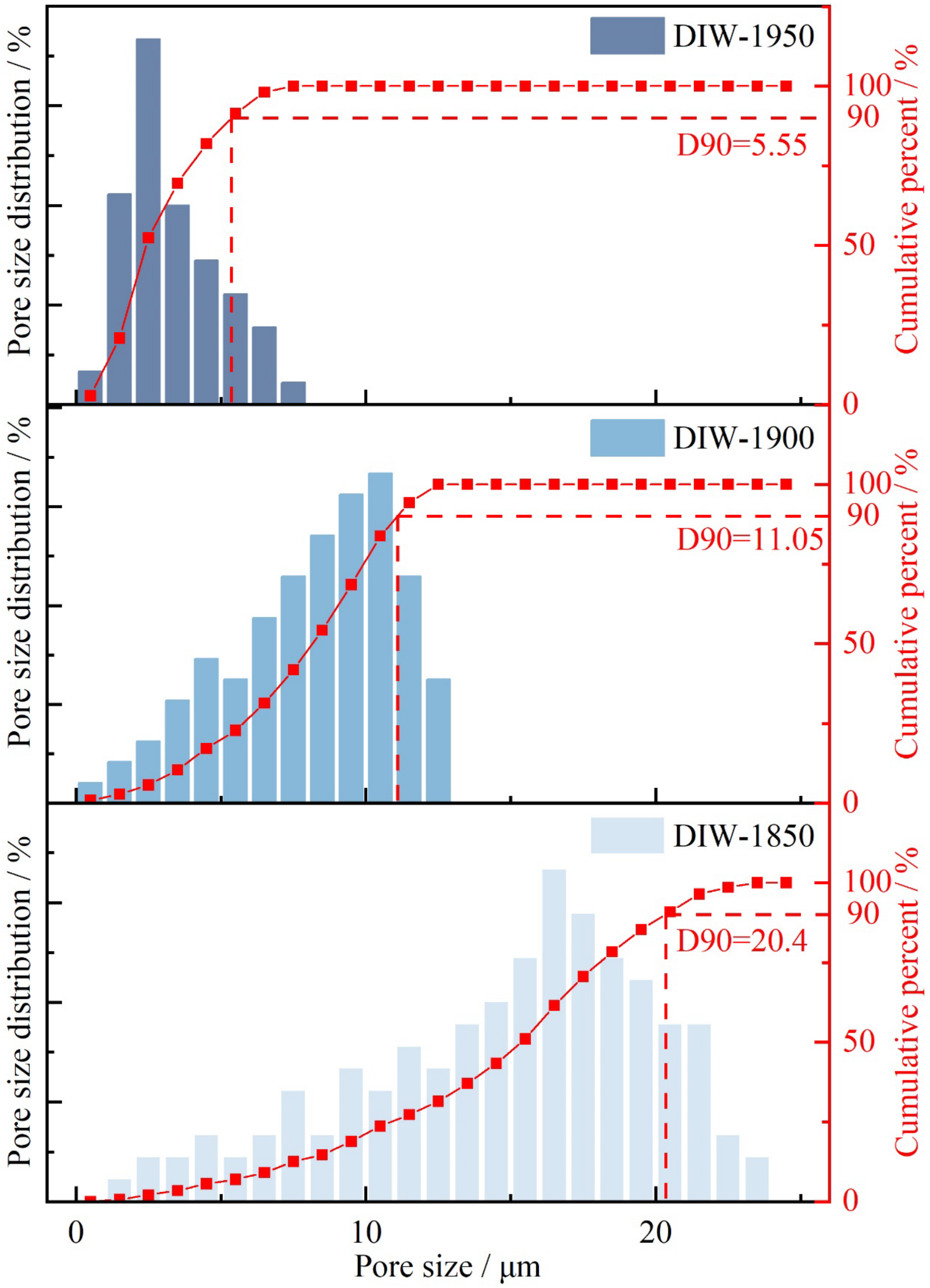

Supplementary Figure 1. Pore size distributions of porous tantalum scaffolds after sintering at different temperatures.

Supplementary Material 2: Computational fluid dynamics (CFD) simulation

The optimal DIW printing condition is when the nozzle moving speed is equal to the ink extrusion speed. The ink extrusion speed is controlled by the plunger feed speed, but owing to the small nozzle structure, experimentally measuring the flow rate of the ink inside the nozzle is difficult. Therefore, in this study, the flow rate at the nozzle was predicted in real time using CFD simulation, providing theoretical guidance for determining the ranges of the nozzle moving speed and plunger feed speed in DIW printing. The CFD simulation was conducted with Workbench 19.0 software.

Based on the experimental setup, a physical model identical to the extrusion module (inlet diameter of 28 mm and outlet diameter of 0.6 mm) was created. Mesh generation was performed using ICEM CFD software, resulting in approximately 270,000 cells with good mesh quality. After the mesh file was imported into Fluent CFD, the material properties of the pre-printing ink within the fluid domain were defined based on previous calculations of the ink density and test results of its rheological characteristics. The specific parameters are shown in Table 1.

Supplementary Table 1 Physical properties of the pre-printing ink

Parameter	Value
Density / kg m^{-3}	16680
Zero shear viscosity / Pa s	75395
Relaxation time / s	21.45
Power-law index	0.369

Before setting the boundary conditions, the flow regime of the pre-printing material in the nozzle (laminar or turbulent) was first determined. This was accomplished by calculating the Reynolds number (*Re*) values at the inlet and outlet and comparing them with the critical Reynolds number (Re_{cr}): if $Re < Re_{cr}$, the flow is laminar; if $Re > Re_{cr}$, the flow is turbulent. The critical Reynolds number is $Re_{cr} = 2320$, and *Re* was calculated using Eq. (1).

$$Re = \frac{\rho v D}{\mu} \quad (1)$$

where ρ represents the density of the pre-printing ink, v is the average flow velocity, D is the equivalent diameter, taken as the barrel diameter, and μ is the viscosity of the pre-printing ink. The parameter values used for calculating the Reynolds number of the pre-printing ink with Eq. (1) are shown in Table 2.

Supplementary Table 2 Parameter values used for calculating the Reynolds number of the pre-printing ink

	ρ / kg m^{-3}	v / m s^{-1}	D / m	μ / Pa s
inlet	1.668×10^{4}	8.333×10^{-6}	2.8×10^{-2}	7.482×10^{4}
outlet	1.668×10^{4}	1.814×10^{-2}	6×10^{-4}	23

The Re values at the inlet and outlet are much smaller than Re_{cr}; thus, the flow regime of the pre-printing ink in the physical model is laminar, and the fluid model was set to laminar flow. Three different plunger feed speeds (0.1, 0.3, and 0.5 mm/min) were applied to calculate the flow velocity variation at the centre of the nozzle outlet and determine the flow velocity distribution of the pre-printing ink within the nozzle structure.

Supplementary Material 3: Characterization

The microscopic morphology of the tantalum powders, green scaffolds and sintered scaffolds was analysed using scanning electron microscopy (SEM) (SU-8010; Hitachi, Japan) with a voltage of 5 kV. The phase compositions of the tantalum powders and the sintered scaffolds were analysed using X-ray diffraction (XRD) (XRD-7000, Shimadzu, Japan) at a scanning speed of 2 ° min^{-1}. The filament diameter of the printed green scaffolds was measured using an optical imaging instrument (VML300, 3D FAMILY, China).

Thermogravimetric analysis (TGA) of the green scaffolds was performed by a simultaneous thermal analyser (TA SDT Q600, Scientific Compass, China) with a prescribed temperature range of 30°C–600°C, a heating rate of 10 °C min^{-1}, and argon gas for the test environment. The shrinkage rate of the samples was calculated by

measuring the linear shrinkage in the length, width, and height directions after sintering using a Vernier calliper. The porosity of the scaffolds was measured using the drainage method. The compressive strength of the sintered scaffolds was evaluated with a universal testing machine (PLD-5; Xi'an Lichuang, China), and an axial load was applied at a rate of 0.5 mm min^{-1} until failure. The stress was defined as the applied force over the area of the sample projected horizontally, and the strain was defined as the displacement over the height of the sample; additionally, the compressive modulus in the quasi-elastic gradient was equal to the slope of the stress–strain relationship in the initial linear regime and was obtained according to the ISO 13314 standard.

Supplementary Material 4: Cytocompatibility and osteogenic assessment

To investigate the effects of scaffolds sintered at different temperatures on cell adhesion and proliferation behaviour, the adhesion and proliferation capabilities of bone marrow mesenchymal stem cells (BMSCs) seeded on the scaffolds were quantitatively assessed. The DIW-1850, DIW-1900, and DIW-1950 scaffolds served as experimental groups. Rat BMSCs at passages 3–5 were cultured in α-minimal essential medium (MEM; HyClone, USA) supplemented with 10% foetal bovine serum (FBS; HyClone, USA) and 1% penicillin–streptomycin (HyClone, USA) under standard conditions (37°C, 5% CO_2). Cell proliferation was assessed by measuring the absorbance at 450 nm using a Cell Counting Kit-8 (Dojindo, Japan) assay. Cells were seeded onto scaffolds (4 mm in diameter) placed in 96-well plates at a density of 1×10^4 cells cm^{-2}. On days 1, 3, and 5 post-seeding, 20 μL of the CCK-8 reagent was added to each well. After 4 h of incubation, the optical density (OD) at 450 nm was measured using a microplate reader (Bio-Rad, USA) and subsequently calculated. To clearly compare the advantages of hierarchical porous structures fabricated by DIW over porous scaffolds produced by mainstream metal additive manufacturing techniques, tantalum scaffolds fabricated by selective laser melting (SLM-Ta) were selected as controls.

Cell viability was further evaluated using live/dead cell staining with propidium iodide (PI; KeyGen Biotech, China). Cells were seeded at a density of 4×10^4 cells per

well onto scaffolds (10 mm in diameter) in 24-well plates. On days 1, 3, and 5 after seeding, the cells were stained with 8 mM PI solution and imaged by confocal microscopy (Nikon, Japan).

To evaluate the osteoinductive activity of the porous tantalum scaffolds, the *in vitro* osteogenic differentiation capacity and osteogenesis-related gene expression were assessed. Rat BMSCs were seeded at a density of 4×10^4 cells mL^{-1} (2 mL $well^{-1}$) onto DIW-1850, DIW-1900, DIW-1950, and SLM-Ta scaffolds placed in 6-well plates. Osteogenic induction medium was prepared by supplementing α-MEM containing 10% FBS with 10 mM β-glycerophosphate (Sigma, USA), 10^{-7} M dexamethasone (Sigma, USA), and 50 μM ascorbic acid (Sigma, USA). After cell attachment, the culture medium in the 6-well plates was replaced with the osteogenic induction medium. After being cultured for 7 and 14 days, the cells were fixed with 4% paraformaldehyde (PFA) and subjected to alkaline phosphatase (ALP) staining using an BCIP/NBT alkaline phosphatase colour development kit (Leagene, China). For the quantitative ALP assay, cells were lysed with RIPA lysis buffer (zhhcbio, China) to extract total protein. ALP activity was determined using a commercial ALP assay kit according to the manufacturer's instructions, and the optical density (OD) was measured at 520 nm. Total protein concentrations were determined using a BCA protein assay kit (Thermo Scientific Pierce, USA), with absorbance measured at 562 nm. The ALP activity was finally presented as OD values at 520 nm normalized to the total protein content. The final data are expressed as U/mg protein, ensuring a fair and accurate comparison across groups [1,2].

Mineralization was evaluated through alizarin red S (ARS) staining. After 21 days, the BMSCs on the substrates were fixed by 4% PFA and stained by 1% ARS (pH 4.2; Sigma, USA). The staining results were observed under an optical microscope. For the quantitative assay, cells were harvested by adding 10% acetic acid solution to each well, followed by neutralization of the supernatant with 10% ammonium hydroxide until the pH reached 4.1–4.5, and the OD of each group was detected at 405 nm for semi-quantitative analysis [3,4]. For each group, the absorbance was measured at 405 nm using a microplate reader, with six independent samples and three replicate wells per

sample.

To further verify the osteogenic properties of the scaffolds, rat BMSCs cultured on the different scaffolds for 7 and 14 days were lysed using TRIzol reagent (TakaRa, Japan) for total RNA extraction. Osteogenesis-related gene expression was subsequently analysed by reverse transcription quantitative polymerase chain reaction (RT-qPCR, Takara, Japan), including Collagen Type I *(Col 1)*, Runt-related transcription factor 2 *(RUNX2),* osteopontin *(OPN),* and osteocalcin *(OCN)*. The sequences of the gene-specific primers for rat osteogenic genes are shown in Table 3. Gene expression levels were normalized to GAPDH as the internal control, and this study used the SLM-Ta scaffold as the control. Relative mRNA expression was calculated using the $2^{(-\Delta\Delta Ct)}$ method. The sequences of the specific primers used in this study, provided by Sangon Biotech, China, are listed in Table 3.

Supplementary Table 3 Primer sequences for rat osteogenic genes

Gene	Forward primer sequence (5'-3')	Reverse primer sequence (5'-3')
Col 1	AAAGATGGACTCAACGGTCTC	CAGGAAGCTGAAGTCATAACCA
RUNX2	CTACTATGGTACTTCGTCAGCG	TTTGGATTTAATAGCGTGCTGC
OPN	GTCCAAGGAGTATAAGCAGAGG	GAAACTCGTGGCTCTGATGTTC
OCN	CTGAGTCTGACAAAGCCTTCAT	TCCAAGTCCATTGTTGAGGTAG
GAPDH	CCCCCAATGTATCCGTTGTG	TAGCCCAGGATGCCCTTTAGT

The bone repair ability of scaffolds was evaluated in a rabbit femoral condyle defect model with 24 healthy male New Zealand rabbits (2.5 ± 0.5 kg, Chengdu Dashuo Experimental Animal Co., Ltd., China) after assessing cytocompatibility and osteogenic capacity. All animal experimental procedures underwent ethical review and approval by the Laboratory Animal Center of Air Force Medical University, in accordance with international ethical standards for scientific research (ethical approval No.: 2021 (kq-009)). The animals were housed under conventional conditions with free access to water and food. The rabbits were assigned to four groups: the DIW-1850, DIW-1900, DIW-1950, and defect-only control groups. Samples were collected at 4

and 8 weeks post-implantation, with 3 rabbits per group per time point.

Bilateral cylindrical bone defects (diameter: 4 mm; depth: 6 mm) were created in the lateral femoral condyle of each rabbit using drilling. Scaffolds were implanted into the defects, ensuring close contact with the host bone. Femoral samples were harvested at 4 and 8 weeks post-implantation. Harvested samples were fixed in 4% paraformaldehyde (MedChemExpress, China), embedded, and sectioned. The sections were immersed in 0.1% formic acid (Fuyugs, China) solution for 3 min, rinsed with ultrapure water, immersed in 20% methanol solution (Fuyugs, China) for 2 h, rinsed again, and then stained with picric acid fuchsin solution (Solarbio, China) for 15 min to perform Van Gieson (VG) staining. Following staining, the sections were dehydrated through an ethanol series, dried, and examined under a stereomicroscope for imaging (Olympus, Japan).

REFERENCES

[1] Depboylu F, Yasa E, Poyraz Ö *et al.* Titanium based bone implants production using laser powder bed fusion technology. *J Mater Res Technol* 2022; 17: 1408–26.

[2] Wang X, Xu S, Zhou S *et al.* Topological design and additive manufacturing of porous metals for bone scaffolds and orthopaedic implants: A review. *Biomaterials* 2016; 83: 127–41.

[3] Zhu L, Luo D, Liu Y. Effect of the nano/microscale structure of biomaterial scaffolds on bone regeneration. *Int J Oral Sci* 2020; 12: 6.

[4] Lu Q, Diao J, Wang Y *et al.* 3D printed pore morphology mediates bone marrow stem cell behaviors via RhoA/ROCK2 signaling pathway for accelerating bone regeneration. *Bioact Mater* 2023; 26: 413–24.
